# The relationship between prior antimicrobial prescription and meningitis: a case–control study

**DOI:** 10.3399/bjgp16X684313

**Published:** 2016-03-11

**Authors:** David Armstrong, Mark Ashworth, Alex Dregan, Patrick White

**Affiliations:** Department of Primary Care and Public Health Sciences, King’s College London, London.; Department of Primary Care and Public Health Sciences, King’s College London, London.; Department of Primary Care and Public Health Sciences, King’s College London, London.; Department of Primary Care and Public Health Sciences, King’s College London, London.

**Keywords:** antimicrobial agents, case-control studies, meningitis, microbiome, primary care

## Abstract

**Background:**

Recent research into the role of the human microbiome in maintaining health has identified the potentially harmful impact of antimicrobials.

**Aim:**

The association with bacterial and viral meningitis following antimicrobial prescription during the previous year was investigated to determine whether antimicrobials have a deleterious effect on the nasopharyngeal microbiome.

**Design and setting:**

A case-control study (1:4 cases to controls) was conducted examining the rate of previous antimicrobial exposure in cases of meningitis and in a matched control group. Data from a UK primary care clinical database were analysed using conditional logistic regression.

**Results:**

A total of 7346 cases of meningitis were identified, 3307 (45%) viral, 1812 (25%) bacterial, and 2227 (30%) unspecified. The risks of viral (adjusted odds ratio [AOR] 2.45; 95% confidence interval [CI] = 2.24 to 2.68) or bacterial (AOR 1.98; 95% CI = 1.71 to 2.30) meningitis were both increased following antimicrobial prescription in the preceding year. Patients who received ≥4 antimicrobial prescriptions in the preceding year were at significantly increased risk of all types of meningitis (AOR 2.85; 95% CI = 2.44 to 3.34), bacterial meningitis (AOR 3.06; 95% CI = 2.26 to 4.15) and viral meningitis (AOR 3.23; 95% CI = 2.55 to 4.08) compared to their matched controls.

**Conclusion:**

There was an increased risk of meningitis following antimicrobial prescription in the previous year. It is possible that this increase was due to an effect of antimicrobials on the microbiome or reflected an increased general susceptibility to infections in these patients.

## INTRODUCTION

The pathogenesis of meningitis is believed to involve microorganisms in the nasopharynx crossing mucosal surfaces into the bloodstream from where they gain access to the subarachnoid space^1^–^8^ but what triggers this transmission is less clear. One significant risk factor is the presence of *Neisseria meningitidis* (the causative organism of meningococcal meningitis) in the nasopharynx^9^; carriage rates as high as 24% have been identified in 19-year-olds.^10^,^11^ How is it that a commensal becomes a pathogen? Disturbance of the patient’s microbiome, the enormous numbers of commensal, symbiotic, and pathogenic microorganisms that colonise the human body, may be a plausible explanation.

In recent years a number of studies have begun to explore the role of the human microbiome — mainly those commensal bacteria inhabiting the gut — in maintaining health.^12^,^13^ The integrity of the microbiome may be disrupted by ingesting antimicrobials and there are suggestions that diseases as diverse as asthma and inflammatory bowel disease may have their pathogenesis in antimicrobial-induced harm, particularly in the early years of life.^14^,^15^ At its most extreme, seen commonly in intensive care units, antimicrobial use is associated with overwhelming infections by resistant organisms due in part to the removal of competition from less resistant bacteria in the patient’s gut.

The role of excessive antimicrobial prescribing in provoking the emergence of resistant bacteria has now become a major concern^16^ but antimicrobials may also affect the microbiome in more subtle ways. It is possible that an antimicrobial prescription could, ironically in view of its traditional therapeutic role, increase meningitis risk either by damaging commensals in the nasopharynx resulting in decreased competitive inhibition of other organisms or by impairing the protective effect of the immune system. The present study aimed to investigate the possibility that antimicrobial overprescribing may be associated with increased risk of bacterial and viral meningitis. The study also explored a potential dose–response relationship between antimicrobial prescribing and risk of meningitis. Finally, the study assessed the possibility that the risk of meningitis may vary among antimicrobial drugs with broad and narrow spectrum of action.

## METHOD

A case-control study was implemented in a large primary care database, the Clinical Practice Research Datalink (CPRD). CPRD currently contains medical records from 685 general practices representing one of the world’s largest electronic databases of anonymised longitudinal data from primary care. The size, patients’ characteristics, and geographical distribution of the CPRD primary care practices are representative of the UK population. The database includes complete records of all drugs prescribed, clinical diagnoses, referral to consultants, hospitalisations, and investigation results during primary care consultations. All prescriptions are computer generated and are automatically part of the patient medical record. The data have been extensively validated for pharmacoepidemiological, clinical, and health service usage research.^17^–^20^ Tate and colleagues provide a detailed description of the CPRD. 21

**How this fits in**

While there is considerable evidence that antimicrobials are effective against bacterial infection there is very limited evidence that they may trigger or increase the risk of subsequent infections. The finding that meningitis was more common following antimicrobial prescription could be the result of antimicrobials lowering the threshold for later infections whether through disturbance of the patient’s microbiome or through other mechanisms. This may be another reason for caution in prescribing antimicrobials.

### Study population

Patients with a recorded diagnosis of meningitis between 1 January 1992 and 31 March 2014 represented the cases. The index date was defined as the first date that a diagnosis of meningitis was recorded. Cases were individually matched with up to four randomly selected controls, on age, sex, family practice, and index date for meningitis (controls were given the index date of the meningitis diagnosis of their matched case). Data were extracted in January 2015.

### Outcomes

Medical diagnostic codes were used to identify new diagnoses of meningitis including viral, meningococcal, other bacterial, and unspecified cases.

### Exposure

The exposure variable included any antimicrobial prescription in the 12 months preceding the diagnosis of meningitis; that is, between the baseline and the index date for meningitis. Information extracted included the name of the specific antimicrobial, the number of antimicrobial prescriptions during the follow-up period, and the time lag from drug prescription to meningitis index date. The risk of meningitis following prescription of narrow spectrum antimicrobials and of trimethoprim was also examined: the former could be less harmful to the microbiome and the latter, in targeting predominantly gram-negative urinary tract infections, may have less effect on the mainly gram-positive bacteria in the nasopharyngeal microbiome.

### Confounders

Several variables associated with infection, meningitis risk, and other comorbidities, were included as covariates. These included matching variables (age, sex, index date, and practice); cardiovascular risk factors including cholesterol, body mass index (BMI), (<18.5, 18.5–24.9, 25.0–29.9, 30.0–34.9, and ≥35 kg/m^2^), lifestyle factors including smoking (never, ex-smoker, and current smoker); alcohol (never, ex-drinker, and current drinker); C-reactive protein (CRP) levels; comorbidity including depression, cancer, renal disease, and chronic obstructive pulmonary disorders (COPD), cardiovascular diseases (including stroke, coronary heart diseases, and diabetes); and co-prescribing including use of antihypertensive drugs, statins, and diabetes treatment. For each confounder, the value closest to the study baseline and before an antimicrobial drug prescription was included. For patients without an antimicrobial drug prescription the value closest to the baseline was selected. Where the closest recorded value for lifestyle and cardiovascular risk factors was >5 years prior to the study baseline, a missing value was recorded.

### Statistical analysis

Case-control studies are open to confounding as the apparent relationship between the exposure and disease may be mediated by some other factor. Therefore the relative exposure figures for important clinical factors, such as smoking^22^, that were available in the database and may be associated with both antimicrobial prescription and the occurrence of meningitis adjusted for. In addition, the relationship between antimicrobial consumption and subsequent meningitis may be confounded by indication such that, for example, those patients with prodromal meningitis and/or with a triggering infection could have consulted the GP and obtained an antimicrobial prescription in the days immediately before the emergence of clinically identifiable meningitis. For this reason the time between antimicrobial prescription and meningitis diagnosis was obtained from the database so that the data for the week immediately preceding diagnosis could be treated as possibly being contaminated by a triggering infection. Identifying the time lag between antimicrobial prescription and meningitis diagnosis also allowed examination of the attenuation over time of any association between these two events. The association between antimicrobial use and the risk of viral meningitis were examined, as viruses may not have been directly affected by antimicrobials but their ecological niche in the nasopharynx may have been changed by antimicrobial use.^23^

Conditional logistic regression was used to determine the adjusted odds ratio (AORs) and 95% confidence intervals (CIs) for the association between antimicrobial exposure and risk of meningitis. Data on patients were extracted from the study baseline (the later of the start of the patient’s record in CPRD or 1 January 1992). Follow-up ended at the earliest of the meningitis index date, date of death, and the end of the CPRD record or 31 March 2014. Analyses estimated the associations between antimicrobial drugs and all-cause and subtypes of meningitis. Separate estimation models were conducted for the association of time lag, number of prescriptions (1, 2, 3, and ≥4), and drug type with the risk of meningitis. Analyses were adjusted for study confounders and for age, sex, family practice and index date for meningitis by matching. Data for lifestyle and vascular risk factors (such as, BMI, cholesterol, blood pressure, smoking, and drinking) were not available for all patients and multiple imputation was used to handle missing data. Following Rothman^24^ and Ridker *et al* 25 the analyses did not adjust for multiple comparisons. Data were analysed using Stata (version 12).

## RESULTS

A total of 7346 cases of meningitis were identified during the study follow-up; 3307 (45%) were recorded as viral, 1812 (25%) as bacterial, and 2227 (30%) were unspecified. Table 1 shows the baseline characteristics of study cases and controls. Overall, patients with meningitis were more likely to be diagnosed with COPD, depression, and renal diseases compared to their matched controls.

**Table 1.** Baseline characteristics for study cases and controls

	**Cases (%), *N* = 7346**	**Controls (%), *N* = 29 384**
Mean age (SD), years	22 (19)	22 (19)
Female	3773 (51)	15 092 (51)

**Body mass index^a^**		
Underweight	156 (2)	730 (2)
Healthy weight	1422 (19)	6113 (21)
Overweight	795 (11)	3030 (10)
Obese	488 (7)	1543 (5)

**Smoking^a^**		
Never	1951 (27)	7658 (26)
Past	417 (6)	1497 (5)
Current	1037 (14)	3890 (13)

**Alcohol use^a^**		
Never	541 (7)	2431 (8)
Past	46 (1)	112 (0)
Current	2156 (29)	8593 (29)

**Cholesterol, mg/dL**		
<5.15	337 (5)	1068 (4)
5.15–6.19	229 (3)	749 (3)
≥6.20	129 (2)	426 (1)

**C-reactive protein, mg/L**		
<1	25 (0)	69 (0)
1–3	119 (2)	265 (1)
>3	239 (3)	450 (2)

**Morbidity**		
Renal disease	262 (4)	615 (2)
Coronary heart disease	75 (1)	238 (1)
Stroke	61 (1)	69 (0)
Diabetes mellitus	99 (1)	286 (1)
Cancer	85 (1)	231 (1)
Depression	945 (13)	2221 (8)
Chronic obstructive pulmonary disease	1144 (16)	3.015 (10)
Chronic inflammation	196 (3)	533 (2)

**Therapy**		
Lipid lowering	132 (2)	463 (2)
Antihypertensive therapy	624 (8)	1658 (6)
Diabetes mellitus treatment	95 (1)	270 (1)

a Percentages do not add up to 100% as for some patients the data were missing. SD = standard deviation.

**Figure 1.**
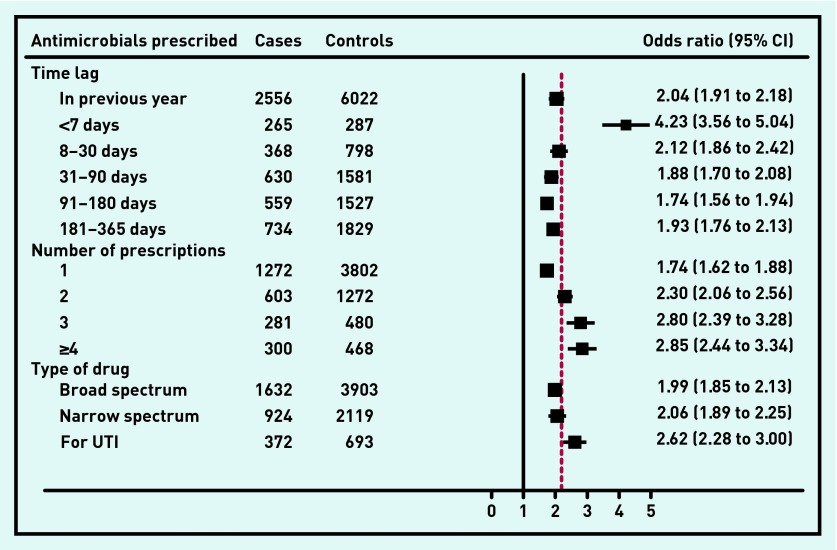
***Association between risk of all-cause meningitis and antimicrobials prescription in the previous 12 months. UTI = urinary tract infection.***

Thirty-five per cent of patients with meningitis had received an antimicrobial prescription in the 12 months preceding their diagnosis compared with 20% of the control group (adjusted OR [AOR] 2.04, 95% CI = 1.91 to 2.18, *P* <0.001). Patients with meningitis were more than four times more likely to have had an antimicrobial prescribed in the 7 days preceding the diagnosis (Figure 1).

Figure 1 also shows the AORs for the risk of meningitis of the number of antimicrobial prescriptions recorded in the previous 12 months for each patient divided into the four categories: 1; 2; 3; ≥4. Patients prescribed ≥4 antimicrobial prescriptions in the previous 12 months had a higher risk of meningitis (AOR 2.85, 95% CI = 2.44 to 3.04, *P* <0.001) compared to those prescribed 0 (*n* = 1) or 1 (AOR 1.74, 95% CI = 1.62 to 1.88) antimicrobial.

**Figure 2.**
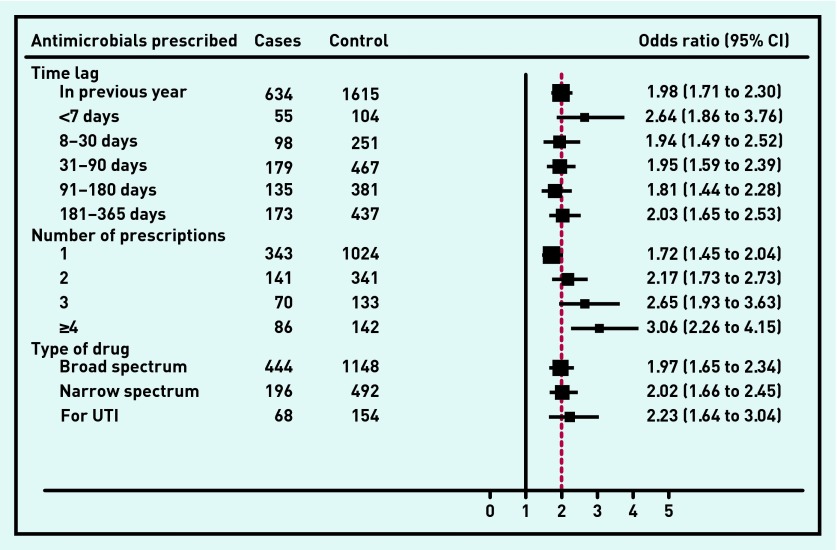
***Association between risk of bacterial meningitis and antimicrobials prescription in the previous 12 months. UTI = urinary tract infection.***

**Figure 3.**
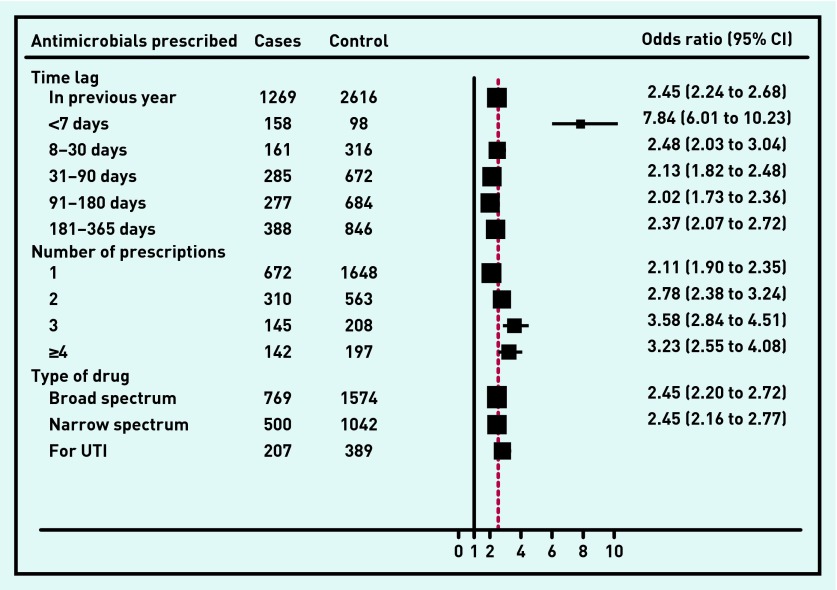
***Association between risk of viral meningitis and antimicrobials prescription in the previous 12 months. UTI = urinary tract infection.***

The relationships between antimicrobial prescriptions in the previous year (and various intervals within that year) and bacterial and viral meningitis are shown in Figures 2 and 3 respectively. The AORs for both types of meningitis were similar though the risk was higher for viral meningitis if antimicrobials had been prescribed in the 7 days immediately preceding diagnosis (AOR 7.84, 95% CI = 6.01 to 10.24, *P* <0.001) than for bacterial meningitis (AOR 2.64, 95% CI = 1.86 to 3.76, *P* <0.001). There were no differences between bacterial and viral meningitis in terms of whether the patient had received a broad or narrow spectrum antimicrobial or trimethoprim prescribed for urinary tract infections.

## DISCUSSION

### Summary

In a large primary care-based population, a strong association was observed between meningitis and antimicrobial prescribing in the previous year. The association was particularly salient with respect to antimicrobials prescribed in the 7 days preceding the meningitis index date which may be the result of prodromal symptoms of meningitis being misdiagnosed, probably as upper respiratory tract infection; indeed, preceding infection/feeling unwell is a recognised risk factor.^26^ Even so, such a ‘prophylactic’ antimicrobial prescription clearly did not have any effect on the subsequent meningitis. Assuming that the prodromal phase of meningitis was no longer than 1 week the association with antimicrobials prescribed in the year preceding the index date (and excluding the previous 7 days) is unlikely to have been confounded by indication.

The original hypothesis that antimicrobials may change the nasopharyngeal microbiome in such a way as to facilitate bacterial or viral meningitis has not been wholly supported by these findings as there was no evidence that the association between antimicrobials and meningitis attenuates over time as may be expected if the microbiome recovered during the year following a prescription. Nor were there reduced risks of meningitis following narrow spectrum antimicrobials and a urinary tract-targeted antimicrobial in the previous year, although there was a dose–response in that patients with ≥4 antimicrobial prescriptions in the previous 12 months had a stronger association with meningitis, particularly bacterial meningitis.

The possible role of antimicrobial damage to the microbiome in increasing the risk of viral meningitis would be more indirect. As there seems to be substantial colonisation of bacteria in the microbiome by viral commensals (bacteriophages), removal of the host bacteria for these viruses by antimicrobial prescription may expose the patient to greater risk of viral pathogen invasion.^23^,^27^,^28^ Alternatively, the association between antimicrobials and meningitis could reflect more generalised immunological suppression or infection susceptibility such as frailty in the patient lasting more than a year which led to presentation to the GP with a variety of infections, including meningitis, for which antimicrobials were prescribed. Indeed, there is evidence that patients with meningococcal disease are more likely to show serological evidence of recent influenza and respiratory syncytial viral infection.^29^,^30^

### Strengths and limitations

The strength of this study lies mainly in the large number of meningitis cases used in the analysis and the likely reliability of the clinical diagnoses and drug prescriptions. The main weakness of this study lies in the difficulty of inferring causality from its case-control design as residual confounding due to unmeasured factors cannot be ruled out. For example, patients with greater general susceptibility to infections, including meningitis, may have presented to their GP more frequently during the previous year and received more antimicrobials. However, the study did adjust for several chronic diseases and related-biomarkers (such as, CRP), commonly used as indicators of frailty.

Meningitis is a diagnosis made in secondary care, most commonly following a lumbar puncture; this diagnosis would then be transcribed into the GP electronic record. It is possible that some diagnoses did not get transcribed into the GP records and/or were transcribed without their specific causal organism being identified. These failures may only minimally impact the present findings. Antimicrobial prescriptions are known to be well recorded in the clinical database (although incidental prescriptions given elsewhere, such as in emergency departments or in walk-in centres would not be recorded) but there is no record of whether or not the patients took the prescribed drugs as indicated. Non-adherence by patients, would, if anything, have led to an underestimate of the AORs associated with antimicrobial use.

Nearly 30% of cases of meningitis were recorded without reference to the specific causative agent. It is possible that correct attribution would alter the size of the estimates observed here, but less so the direction of association. The similarity between viral and bacterial meningitis in their association with previous prescriptions of antimicrobials makes it unlikely that this limitation has made an important difference to the observed relationships. A further weakness of this study is that it was not possible to determine the precise indication for the antimicrobial prescriptions recorded and so it cannot be investigated whether the association between these prescriptions and the diagnosis of meningitis is explained by the indication for the prescription or is independent of that indication. The lack of difference between the classes of antimicrobials examined and their association with meningitis would suggest that the indication was not important.

### Comparison with existing literature

There is evidence that infections in patients who have received antimicrobials in the subsequent 6 months are more likely to be caused by resistant organisms^31^ possibly as a result of decolonisation of antimicrobial sensitive organisms within the microbiome. There is also emerging evidence that disruption of the microbiome in the first year of life has a long-term effect on the risk of diseases such as asthma.^32^

### Implications for research and practice

While the exact mechanism for the association between antimicrobial prescription and subsequent meningitis cannot be determined by the case control design used in this study the size of that association merits further investigation. It also adds another reason for caution in antimicrobial prescribing in general practice to the existing concerns about antimicrobial resistance.
